# Enteric Fever: A Slow Response to an Old Plague

**DOI:** 10.1371/journal.pntd.0004597

**Published:** 2016-05-12

**Authors:** Carlos Franco-Paredes, M. Imran Khan, Esteban Gonzalez-Diaz, Jose I. Santos-Preciado, Alfonso J. Rodriguez-Morales, Eduardo Gotuzzo

**Affiliations:** 1 Hospital Infantil de México, Federico Gómez, México D.F., México; 2 Phoebe Putney Memorial Hospital, Albany, Georgia, United States of America; 3 Precision Developmental Research and Advocacy Consultants, Karachi, Pakistan; 4 Hospital Civil de Guadalajara, “Fray Antonio Alcalde”, Guadalajara, México; 5 Unidad de Medicina Experimental, Facultad de Medicina, Universidad Nacional Autónoma de México, Hospital General de México, México, D.F., México; 6 Public Health and Infectious Disease Research Group, Facultad de Ciencias Médicas, Universidad Tecnológica de Pereira, Pereira, Risaralda, Colombia; 7 Instituto de Medicina Tropical, Alexander Von Humboldt, Universidad Peruana Cayetano Heredia, Lima, Peru; University of California San Diego School of Medicine, UNITED STATES

## The Global Burden of Disease Caused by *Salmonella enterica*

**Fig 1:**
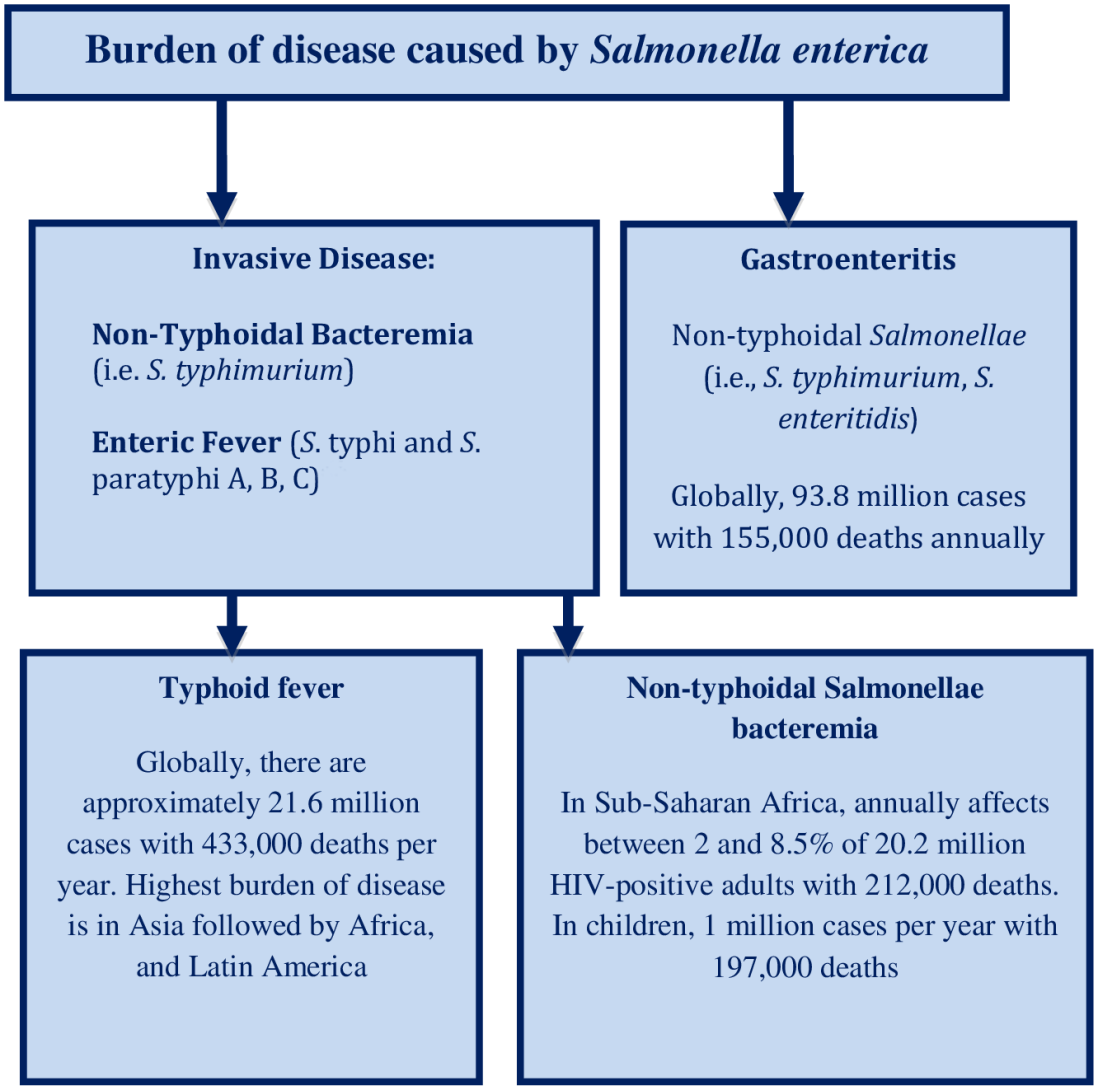
Global estimates of disease caused by *Salmonella enterica* including enteric fever (typhoid fever and paratyphoid fever), non-typhoidal salmonellosis, and gastroenteritis.

Man is irremediably embedded in nature with complex interactions with all living organisms. Historically, the establishment of contemporary human societies has been influenced by our coexistence with other microorganisms living in highly interconnected habitats and ecologies. As a result, with the progression from unicellular to multicellular life, bacteria have coexisted with humans. In this biological journey, while there are important benefits provided by bacterial guests to the human host living in complex relationships and becoming part of their microbiome, some organisms are able to cause a wide spectrum of diseases. Among the large Enterobacteriaceae family, the genus *Salmonella*, a pathotype of *Escherichia coli*, is one example. *Salmonella* is further classified into *S*. *enterica* and *S*. *bongori* serotypes based on its lipopolysaccharide cell wall (somatic O antigen), its flagellar (H antigen), and its surface Vi antigen (present only in *S*. *typhi*, *S*. Paratyphi C, *Citrobacter freundii*, and *S*. Dublin) [1]. *S*. *enterica* subspecies I, one of the six subspecies of *S*. *enterica*, is a major contributor to human disease (Fig 1) [2]. This group of pathogens includes those frequently causing gastroenteritis, such as *S*. Typhimurium, those causing invasive disease in the forms of bacteremia, such as *S*. Choleraesius, or the typhoidal *Salmonella* species causing enteric fever, including *S*. *typhi* (typhoid fever) and *S*. Paratyphi A, B, and C (paratyphoid fever) [1,2].

Currently, despite major efforts in preventing and treating cases of enteric fever, millions of new infections (approximately 21 million new cases per year) of typhoid and paratyphoid fevers occur in many areas where poor sanitation and unsafe food and water access occurs frequently and among travelers to endemic areas [2]. Furthermore, combined morbidity and mortality caused by *S*. *enterica* serovars globally cause one million deaths annually (Fig 1) [1,2]. These figures are likely underestimated due to (a) limited surveillance activities in the most affected areas, (b) diagnostic challenges such as low sensitivity of blood cultures and only a few institutions in highly endemic settings performing blood cultures, and (c) underlying healthcare inequalities resulting in low health-seeking behavior among populations at the highest risk of becoming ill by this bacterial pathogen.

## The Undefined Burden of Disease of Enteric Fever

Despite the facts that *S*. *typhi* was isolated more than 130 years ago and typhoid vaccines have been available since 1889, we have failed to create a momentum to reduce the medical impact of this pathogen. The bacteriologic history of typhoid fever began in 1880 with Carl Joseph Eberth and Robert Koch identifying the bacillus in cadaveric tissues independently [3–6] and Georg Gafky culturing the bacillus [4]. The term “typhoid fever” was coined by the French physician Pierre Charles Alexander Louis [5], who described the clinical and pathological aspects of the disease associated with the “typhoid pattern” of mental fogginess and persistent fever mimicking the symptoms caused by typhus [6]. The term “enteric fever” was later coined by the pathological identification of a pattern of hyperplasia of Peyer’s patches with abundant typhoid bacilli in the distal ileum in association with mesenteric lymphadenopathy in patients with a clinical picture compatible with typhoid or paratyphoid fever [6]. There are many prevailing challenges in reducing the burden of diseases caused by enteric fever, including: insufficient and incomplete surveillance activities, inappropriate allocation of public health resources towards priority areas, and a lack of consensus on vaccination strategies in highly endemic areas (Table 1). Additionally, current estimates of enteric fever do not incorporate information on severe and complicated forms of the disease and the impact of antimicrobial resistance to clinical outcomes, further complicating control efforts [7–9].

**Table 1:** Challenges in reducing the burden of disease of enteric fever (typhoid fever and paratyphoid fever).

Currently available burden of disease estimates of enteric fever have demonstrated some improvement in the overall burden of typhoid and paratyphoid fevers^a^. However, these estimates do not include factors such as disease severity and complications; hence, these estimates have a limited impact implementing effective public health policies such as vaccination efforts
In some endemic settings, increased urbanization and population migration from rural settings into unregulated urban and peri-urban settlements lead to increased risk factors to acquire enteric fever (poor sanitation and hygienic practices)
The risk of acquisition of typhoidal salmonellosis has increased in many endemic settings, most notably due to: • Slow socioeconomic development combined with poor governance and lack of political commitment, resulting in minimal improvements in water and sewage management systems, which is linked to poor sanitary practices, • Natural catastrophes such as earthquakes or floods, and • Civil strife leading to destruction of infrastructure, displacement of populations, and resettlement into refugee camps
Fluoroquinolones prescribed in outpatient settings (prescription and nonprescription) in settings endemic for enteric fever have attenuated clinical severity and rates of hospitalization. It is not clear if the rate of complications associated with enteric fever has decreased. These practices, in turn, have promoted increasing rates of antimicrobial resistance, thus limiting the clinical utility of fluoroquinolones

^a^ According to the Global Burden of Disease 2013 Study, the global incidence of 188 countries for typhoid fever was 13,685x1,000 in 1990 and 10,955x1,000 in 2013, representing a 20% improvement (33% improvement in years lived with disability [YLD]). Regarding paratyphoid fever, there has been a 28% decrease in the global incidence when comparing 1990 to 2013 (with a concomitant 39% improvement in YLD) [Global Burden of Disease Study 2013 Collaborators. Global, regional, and national incidence, prevalence, and years lived with disability for 301 acute and chronic diseases and injuries in 188 countries, 1990–2013: a systematic analysis for the Global Burden of Disease Study 2013. Lancet 2015; 36(9995): 743–800]

There are also major gaps in current estimates on the burden of disease from Latin America and Africa [1,9]. Indeed, a large number of cases are reported yearly among most Latin America countries. According to data from the Ministry of Health, 5,960 cases of enteric fever have been reported in Colombia between 2009 and 2013 (3,643 of typhoid fever and 2,467 of paratyphoid fever) (Personal communication, Alfonso Rodriguez-Morales). In Peru, while there has been an important reduction in cases of typhoid-associated intestinal perforations, *S*. *typhi* remains a common blood-borne pathogen identified in hospital settings with only a limited number of cases of *S*. Paratyphi (Personal communication, Eduardo Gotuzzo). In sub-Saharan Africa, a region that was once considered to be typhoid-free, recent reports are raising concerns about the substantial impact of *S*. *typhi* and the non-typhoidal salmonellosis associated with invasive disease [9]. In Latin America and sub-Saharan Africa, epidemiologic trends in urban and rural settings as well as populations at risk of developing severe disease, complications, and mortality require systematic efforts to delineate the associated burden of disease at a population-based level [9].

Across South and Southeast Asia, enteric fever remains the most significant *S*. *enterica* clinical manifestation [1,9]. In this region, most of the data on the burden of disease of enteric fever stem from vaccine clinical trials and active population community-based surveys in which the natural history of enteric fever is modified by active case identification and effective management. Conversely, information on severe forms of the disease based on hospital-based case series also provides an incomplete perspective on the burden of disease. In Asia, research conducted on enteric fever over the last decade has uncovered a high disease incidence of enteric fever with elevated morbidity, particularly in the pediatric age group (<5 years of age), which was once considered an enteric fever-free age group. Recent reports also demonstrate a rising number of cases of *S*. Paratyphi in some areas in Asia [1]. Understanding of the overall burden of disease caused by typhoid fever in terms of disease severity, antimicrobial resistance, associated complications (e.g., encephalopathy, intestinal perforation, and others), and mortality in Asia and Africa remains to be fully elucidated.

To address the limitations of hospital-based reports of enteric fever to assist in estimating the burden of disease at a population level, community-based active surveillance efforts have been conducted in an attempt to identify cases of typhoid fever and paratyphoid fever occurring at the community level through active case finding [8]. An important shortcoming of this approach is that, similar to what occurs during surveillance carried out at the time of vaccine clinical trials, active community-based surveillance focuses on early detection of cases and thus most cases are effectively treated. As a result, the natural history of severe disease, hospitalization rates, and associated complications that otherwise occur in the community are not captured, thus offering the impression of enteric fever manifesting only as a mild disease. In fact, some prospective community-based studies of febrile illness and blood culture-confirmed typhoid fever from hospital-based series have found few complications and minimal long-term sequelae with a limited case fatality from typhoid fever [10–13].

There is an urgent need to assess burden of disease estimates of enteric fever at a population-based level to provide a clear narrative to the world of the scale of the problem. In this regard, there is clear evidence that, in many endemic settings, enteric fever is associated with substantial morbidity [10,11]. For example, in Vietnam, 15.5% of all culture-confirmed enteric fever cases were classified as severe disease [10]. In addition, typhoid fever was found to be the second leading cause of perforation peritonitis in a case series in Delhi, India [12], and a systematic review found a mortality rate of 15.5% in hospitalized cases of intestinal perforation [13,14]. Therefore, a hidden burden of disease of enteric fever remains to be uncovered among disenfranchised populations in affected areas where this pathogen continues to inflict immense human suffering, cause a majority of health spending by affected families and medical institutions, and be a socially-sculpted infectious disease.

## The Way Forward to Reduce the Burden of Enteric Fever

Typhoid fever is a life-threatening infectious disease that, paradoxically, can be prevented. As huge investments and infrastructure are needed for developing sanitation and safe water systems in highly endemic areas for enteric fever, and these improvements may take years to develop, there is an important need to identify interventions that will contribute to establishing a global strategy for control or elimination of enteric fever.

From a disease surveillance perspective, there is a need for focusing on severity of the disease, its associated complications, and clinical outcomes, as such estimates may effectively translate into effective public health action. Once available, updated burden of disease estimates must be packaged into messages targeting leaders and policy-makers in highly affected areas. Interventions designed to reduce the burden of disease of enteric fever, uncovered by improved surveillance activities and burden of disease estimates, must consider multiple approaches to reduce the medical and societal cost of enteric fever, including: (a) introducing vaccines into routine national immunization programs of highly endemic areas [14,15]; (b) effective medical treatment of cases and their associated complications; and (c) water sanitation strategies [8]. At this point in time, currently available formulations of typhoid vaccines are mostly used for travelers visiting endemic areas or in the private market of highly endemic settings. Regretfully, these vaccines are not readily available to the populations that need them the most [14].

A global strategy to decrease the overall medical and social impact of enteric fever offers an opportunity to decrease the impact of an infectious disease that has afflicted humankind for millennia and has a direct link with pervasive health inequities.
